# Supplementary material for: Hierarchical fluctuation shapes a dynamic flow linked to states of consciousness
Source: Nat Commun. 2023 Jun 5;14:3238. doi: 10.1038/s41467-023-38972-x (PMC10241811; doi:10.1038/s41467-023-38972-x)
Supplement: Supplementary file 1 — Supplementary Information [file 41467_2023_38972_MOESM1_ESM.pdf]

Supplementary Materials

A note on global signal and physiological noise

Global signal regression

We assessed the potential impact of global signal regression (GSR) on the relationship between the hierarchical index and global states of consciousness. In the case of dexmedetomidine-induced sedation and sleep data, we incorporated the averaged signals within the brain mask as an additional regressor in the denoising procedure embedded in BRANT[1]. For the HCP resting-state data, we performed global signal regression on the ICA-FIX signals, taking into account all 91282 grayordinates. We applied the same procedure for calculating the hierarchical index in three paradigms related to states of consciousness. As a result, there was no significant deviation from our key findings presented in Fig. 2 in the main text: i) The hierarchical index with GSR distinguished the sedation state from wakefulness/recovery at the individual level (wakefulness versus sedation: $t = 6.72$, $P < .01$; recovery versus sedation: $t = 3.20$, $P < .01$). ii) The hierarchical index with global signal regression significantly decreased across the resting-state scanning in 34.5% of the individual runs, whereas only 0.7% significant increased (uncorrected $P < .05$). iii) The hierarchical index with global signal regression captures the temporal variation in sleep stages in each of six volunteers (For subject 1: $r = 0.76$, $P < .01$; For subject 2: $r = 0.89$, $P < .01$; For subject 3: $r = 0.76$, $P < .01$; For subject 4: $r = 0.76$, $P < .01$; For subject 5: $r = 0.85$, $P < .01$; For subject 6: $r = 0.88$, $P < .01$; Pearson correlation).

Head Motion

Several different strategies for head motion correction have been performed in fMRI preprocessing pipelines. In the BRANT-based preprocessing pipeline (e.g., anesthesia and sleep fMRI datasets), Friston's 24 head motion parameters[2] (3 rotation and 3 translation parameters, 6 parameters one time point before, and the 12 corresponding squared items) were considered in the nuisance regressors; In the HCP pipeline, the ICA components classified as movement-related issues can be removed in the ICA-FIX procedure. To evaluate the contribution of head motion, we conducted the following additional analyses:

Dexmedetomidine-induced Sedation. We did not observe a systematic sedation effect on volunteers' head motion during the scanning. There was no significant difference in mean framewise displacement (FD) between awake and sedation states ($P = .41$; paired T-test), and there was mild difference between recovery and sedation states ($P = .02$; Paired T-test). To control the effects of larger motion frames, we conducted an additional scrubbing strategy (FD > 0.5 mm) before the nuisance regression and bandpass filtering. 8% frames were removed on average and at least over 50% frames were kept for each scan. As a result, similar differences can be reliably detected based on the

hierarchical index with motion scrubbing strategy (wakefulness versus sedation: t = 6.88, $P < .01$; recovery versus sedation: t = 3.25, $P < .01$).

HCP resting-state data. First, we selected 100 HCP individuals with largest tendency of the decreasing hierarchical index during the 4 resting-state scanning ($r_{average}$= -0.57 during 24 partitions per run). No significant difference in mean FD measurement was observed between these selected individuals and others, which indicates that the temporally decreasing hierarchical index cannot be explained by overall motion level. Next, the partial correlation between hierarchical index and scanning time (across 24 temporal partitions) was performed each run to control the effects of FD. Compared to the reported results, the partial correlation controlling head motion has a similar temporal drifting (36.8% of the individual runs decreased and 1.3% significant increased, uncorrected $P < .05$).

Sleep. Corresponding to our temporal partition, we averaged FD measurement within each 150 s time window across whole fMRI scanning for 6 volunteers. A significant but inconsistent correlation between mean FD and sleep stages (For subject 1: $r = 0.34$, $P = .023$; For subject 2: $r = 0.39$, $P < .01$; For subject 3: $r = 0.29$, $P > .05$; For subject 4: $r = -0.65$, $P < .01$; For subject 5: $r = 0.69$, P < .01; For subject 6: $r = -0.48$, $P < .01$; Pearson correlation) was observed. Such result indicates an influence of global brain states on head motion; however, the inconsistence excluded the possibilities of a systematic bias on our key result, which showed highly consistent across subjects. Subsequently, a partial correlation was performed between the hierarchical index and sleep stages, controlling for the effect of mean FD, and the associations were still considerable (For subject 1: $r = 0.76$, $P < .01$; For subject 2: $r = 0.78$, $P < .01$; For subject 3: $r = 0.89$, $P < .01$; For subject 4: $r = 59$, $P < .01$; For subject 5: $r = 0.65$, $P < .01$; For subject 6: $r = -0.84$, $P < .01$; Partial correlation). We also tested an additional scrubbing approach in the preprocessing (see above), and 7% frames were removed on average for each volunteer. As a result, the correlation between sleep stages and the hierarchical index with motion scrubbing strategy (For subject 1: $r = 0.76$, $P < .01$; For subject 2: $r = 0.78$, $P < .01$; For subject 3: $r = 0.89$, $P < .01$; For subject 4: $r = 59$, $P < .01$; For subject 5: $r = 0.65$, $P < .01$; For subject 6: $r = -0.84$, $P < .01$; Pearson correlation) was comparable to our reported results, excluding the effects of large motion frames.

Physiological Fluctuation.

To evaluate the influence of respiratory and cardiac cycles, we derived physiological fluctuations based on pulse oximeter signals in HCP data. Raw physiological signals (*Physio_log.txt) were further processed using the PhysIO toolbox (https: //github.com/translationalneuromodeling/tapas/tree/master/PhysIO). Specifically, RETROICOR[3] was applied to model the relationship between the BOLD signal and the phase of the filtered respiratory and cardiac signal using 4th order and 3rd order Fourier series, respectively. This resulted 14 regressors for each run: 8 regressors modelling the respiratory phase (breathing motion) and 6 regressors modelling the cardiac phase (cardiac pulsatility). The 14 regressors were included in a regression model using fMRI signals after ICA-FIX and the

residuals were further filtered in the frequency band of 0.01-0.08 Hz. As a result, the hierarchical index (with physiological cycles regressed) significantly decreased across the resting-state scanning in 35.2% of the individual runs, whereas only 1.7% significant increased (uncorrected $P < .05$).

Supplementary Figure 1

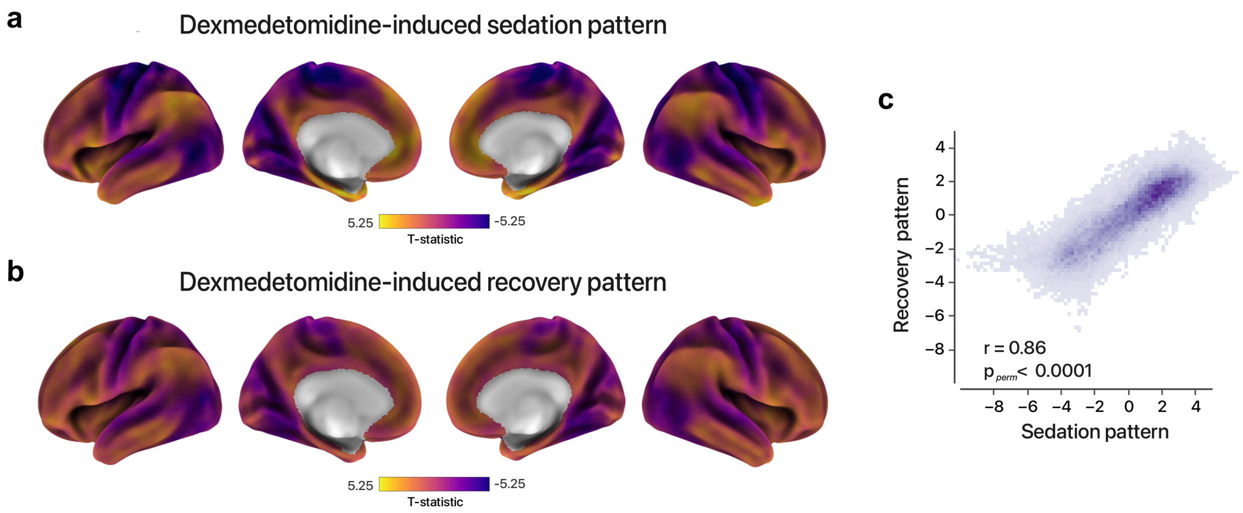

Fig S1. a-b, Cortex-wide unthresholded *t*-statistical map of dexmedetomidine-induced sedation (a) and recovery (b) effects. For the purposes of visualization as well as statistical comparison, the map was projected from the MNI volume into a surface-based CIFTI file format (59412 vertices). **c,** Sedation pattern (wakefulness versus sedation) is highly correlated with the recovery pattern (recovery versus sedation), $r = 0.86$, $P_{perm} < .0001$, two-sided Spearman correlation.

Supplementary Figure 2

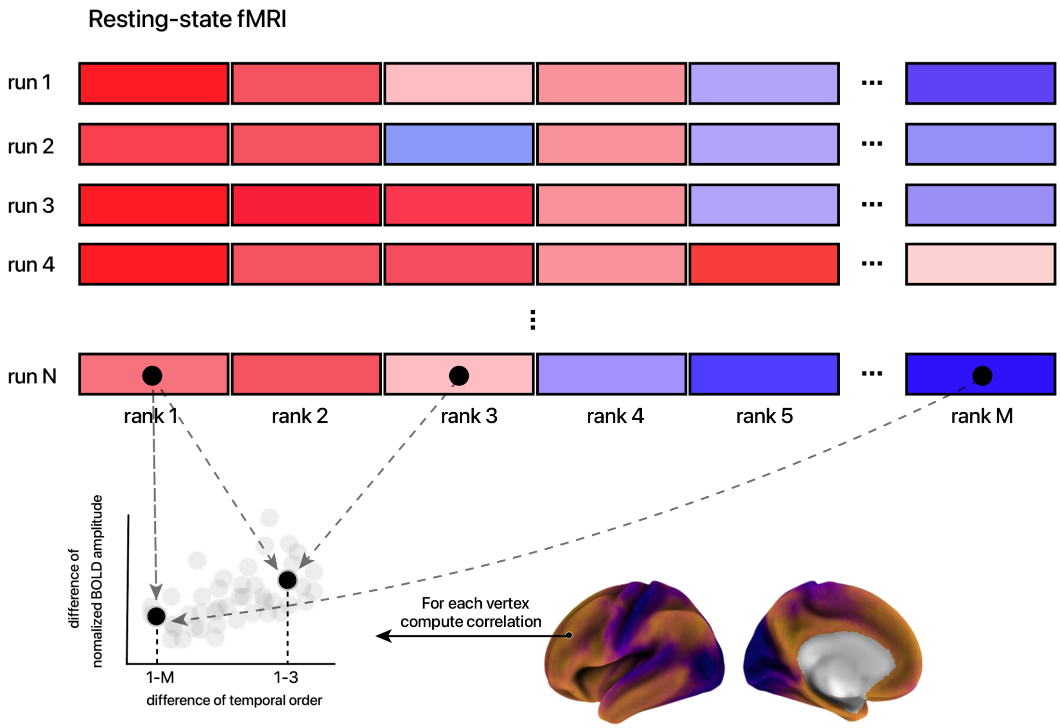

Fig S2. A schematic diagram illustrating the method used to generate the decrease in vigilance pattern from resting-state fMRI data. Four resting-state runs were performed for each individual, with each run consisting of 1200 frames divided into 24 non-overlapping windows. Within each run, every two windows were paired as a sample, with the greater the temporal distance between the two windows, the more likely a decrease in vigilance level was assumed, i.e., for each cortical vertex, the difference in temporal order and the difference in normalized BOLD amplitude were correlated across all pairs of windows and runs.

Supplementary Figure 3

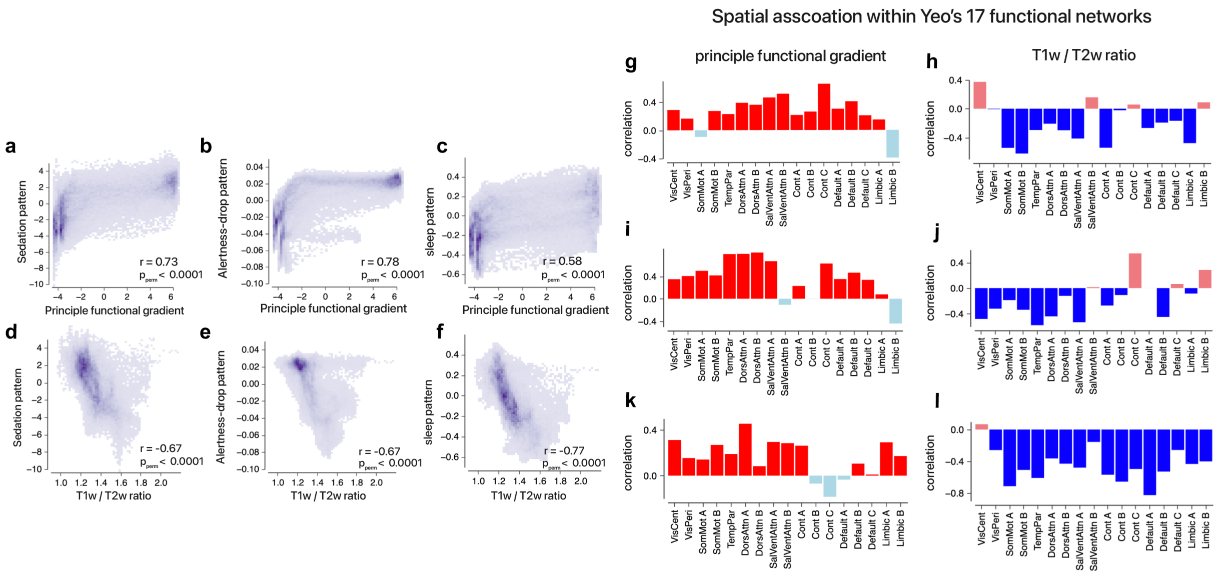

Fig S3. a-c, T1w/T2w ratio map captures spatial variation in the conscious-related patterns across surface area (Wakefulness versus Sedation: r = -0.67, P_{perm} < .0001; HCP vigilance: r = -0.67, P_{perm} < .0001; Sleep: r = -0.77, P_{perm} < .0001, Spearman correlation with permutation-based P value). **d-f,** Rank correlation coefficient between principal functional gradient and conscious-related patterns within each of 17 Yeo's functional network (VisCent n = 3861, VisPeri n = 3368, SomMot A n = 5845, SomMot B n = 4959, TempPar n = 2031, DorsAttn A n = 3468, DorsAttn B n = 3485, SalVentAttn A n = 4945, SalVentAttn B n = 3192, Cont A n = 3488, Cont B n = 3473, Cont C n = 1365, Default A n = 4776, Default B n = 4589, Default C n = 1396, Limbic A n = 2347, Limbic B n = 2053). **g-i,** Rank correlation coefficient between T1w/T2w ratio map and conscious-related patterns within each of 17 Yeo's functional network.

Supplementary Figure 4

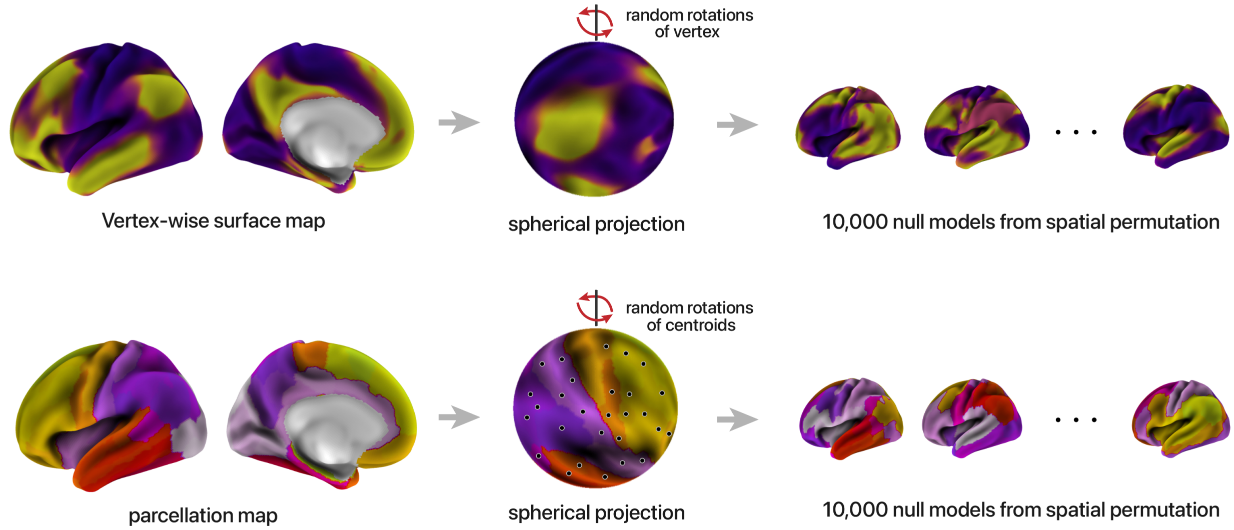

Fig S4. The generation of 10,000 null models accounting for the effects of spatial autocorrelation. Top: vertex-wise surface-based spatially permutation models on the Alexander-Bloch method[4]. Bottom: ROI-level surface-based spatially permutation models on the Vázquez-Rodríguez method[5].

Supplementary Figure 5

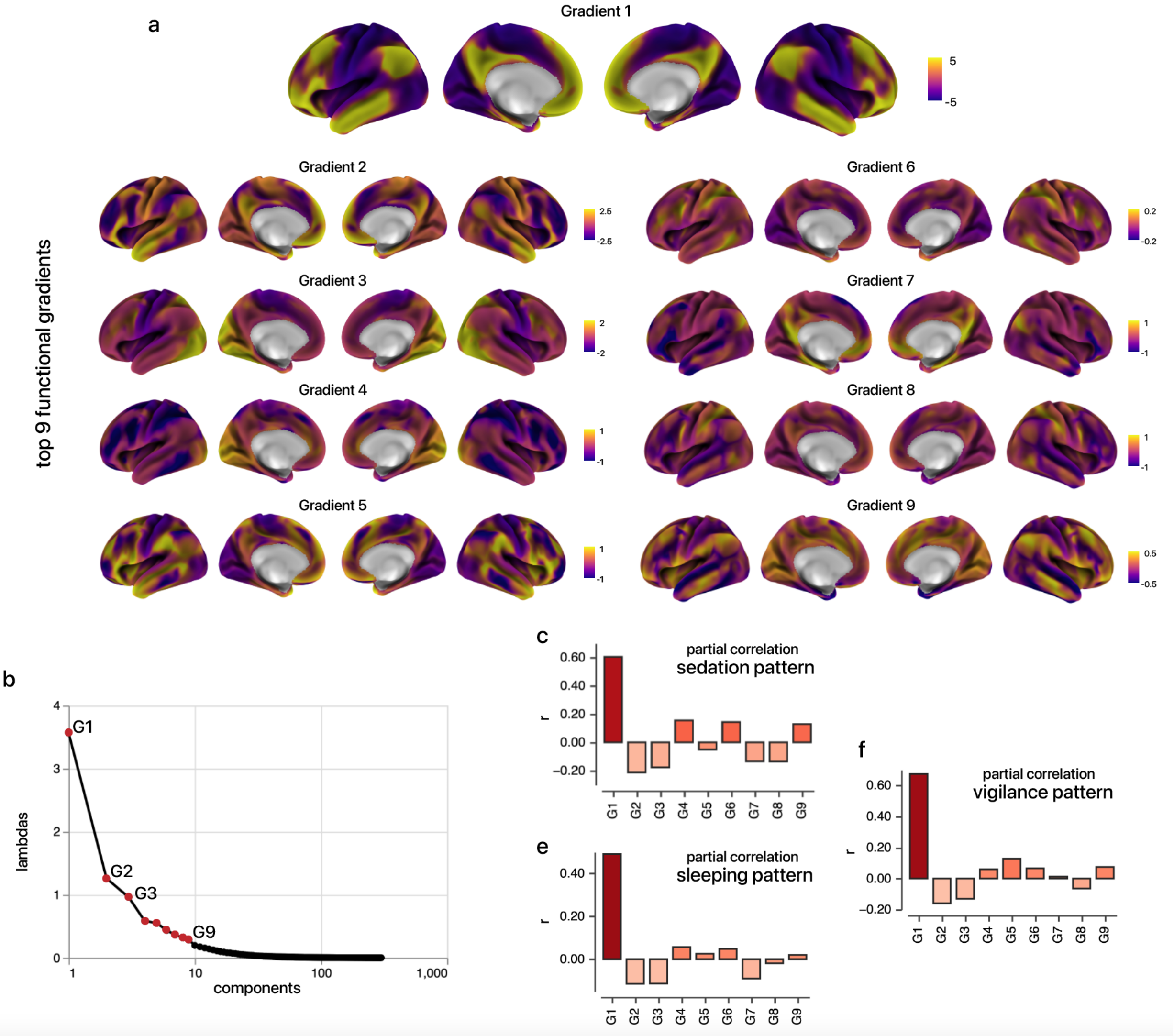

Fig S5. Top nine functional gradients and spatial signature of cortex-wide BOLD amplitude relating to anesthetic effects, sleep, and vigilance. **a**, Visualization of 1st to 9th functional gradients of human functional connectome during resting-state. The principal gradient ranges from unimodal to multimodal networks; the 2nd gradient ranges from visual to somatosensory/motor cortex; the 3rd gradient ranges from default mode network to attention networks; 4th gradient ranges from visual to attention networks; 5th gradient ranges from visual, somatosensory/motor to auditory regions; the subsequent gradients become increasingly complex. **b**, The top 300 eigenvalues from diffusion embedding of dense functional connectome. **c-f**, Partial correlations between nine cortical functional gradients and consciousness-related maps (top left, c: sedation pattern; bottom left, e: sleep pattern; right, f: vigilance decrease pattern). Higher association value corresponds to higher saturation of red.

Supplementary Figure 6

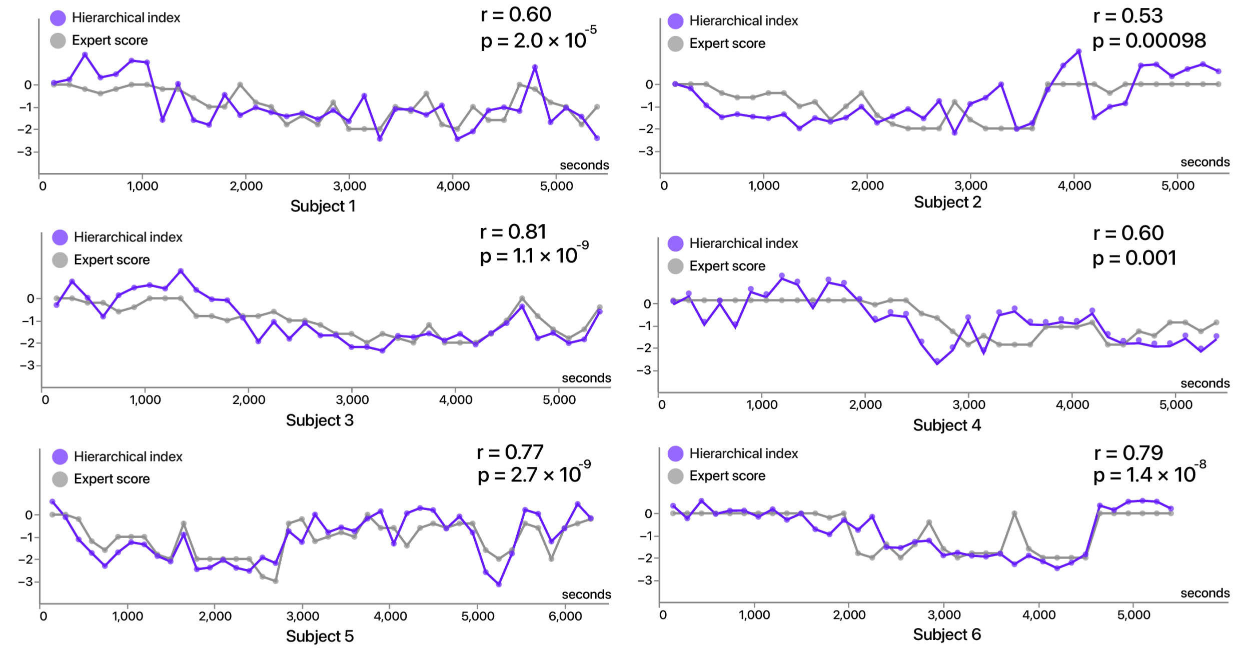

Fig S6. Hierarchical index captures the temporal variation in sleep stages in independent datasets (gray line: expert scores; blue line: hierarchical index; two-sided unadjusted Pearson correlation). The vertical axis represents four sleep stages (wakefulness = 0, N1 = -1, N2 = -2, slow-wave sleep = -3), with time shown on the horizontal axis; for visualization, we normalized the hierarchical index over time and added the mean of the corresponding expert score.

Supplementary Figure 7

Dynamic global signal topology analysis in another 100 unrelated individuals

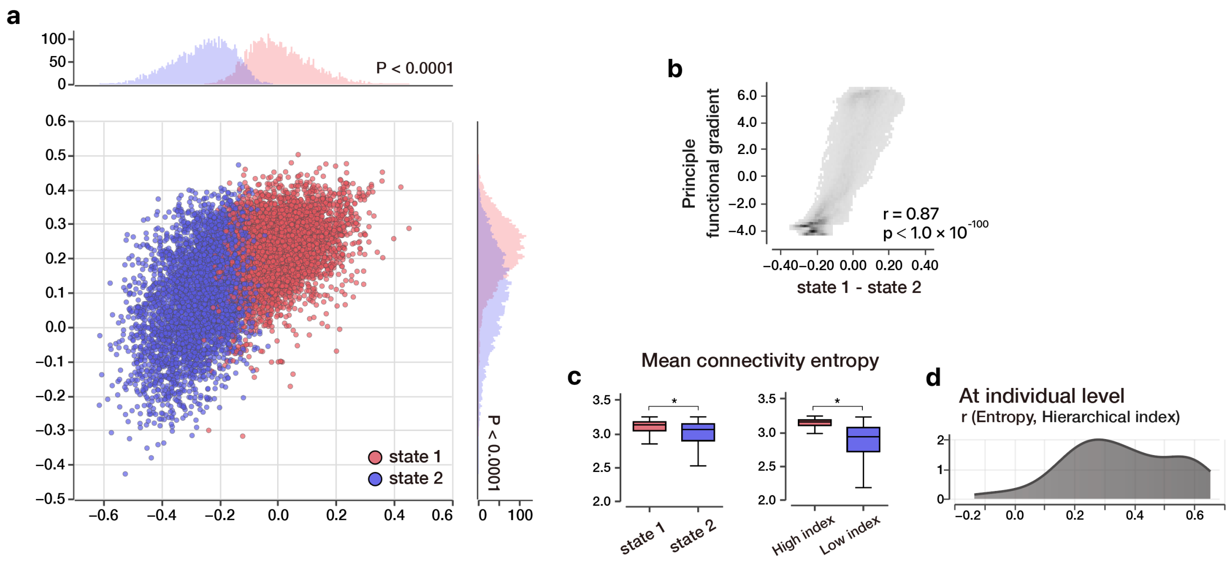

Fig S7. Validation of dynamic GS topology analysis based on another 100 independent subjects. a, 2-cluster solution of GS topology in 9,600 time windows. State 1 has a significantly higher hierarchical index (P < .0001, two-sided two-sample t-test) and hierarchical similarity of the GS topology (P < .0001, two-sided two-sample t-test) than State 2. Two variables are moderately correlated (r = 0.59, P < .0001, two-sided unadjusted Spearman correlation). **b,** Difference in GS topology between State 1 and State 2 spatially recapitulates principal functional gradient (r = 0.87, $P < 1\times10^{-100}$, two-sided unadjusted Spearman correlation). **c,** Left: Higher overall connectivity entropy in State 1 than in State 2 ($P = 4.3\times10^{-43}$, $n_{state\ 1} = 4779$, $n_{state\ 2} = 4821$, two-sided two-sample t-test). Right: Higher overall connectivity entropy in states with high hierarchical index (top 20% versus bottom 20%; $P < 1\times10^{-100}$, $n_{high} = 1920$, $n_{low} = 1920$, two-sided two-sample t-test). *P < .0001. In each box plot, the midline represents the median, and its lower and upper edges represent the first and third quartiles, and whiskers represent the 1.5 × interquartile range. **d,** Distribution of Pearson's correlation between the hierarchical index and mean connectivity entropy across 96 overlapping windows (24 per run) in another 100 individuals (mean r = 0.381).

Supplementary Figure 8

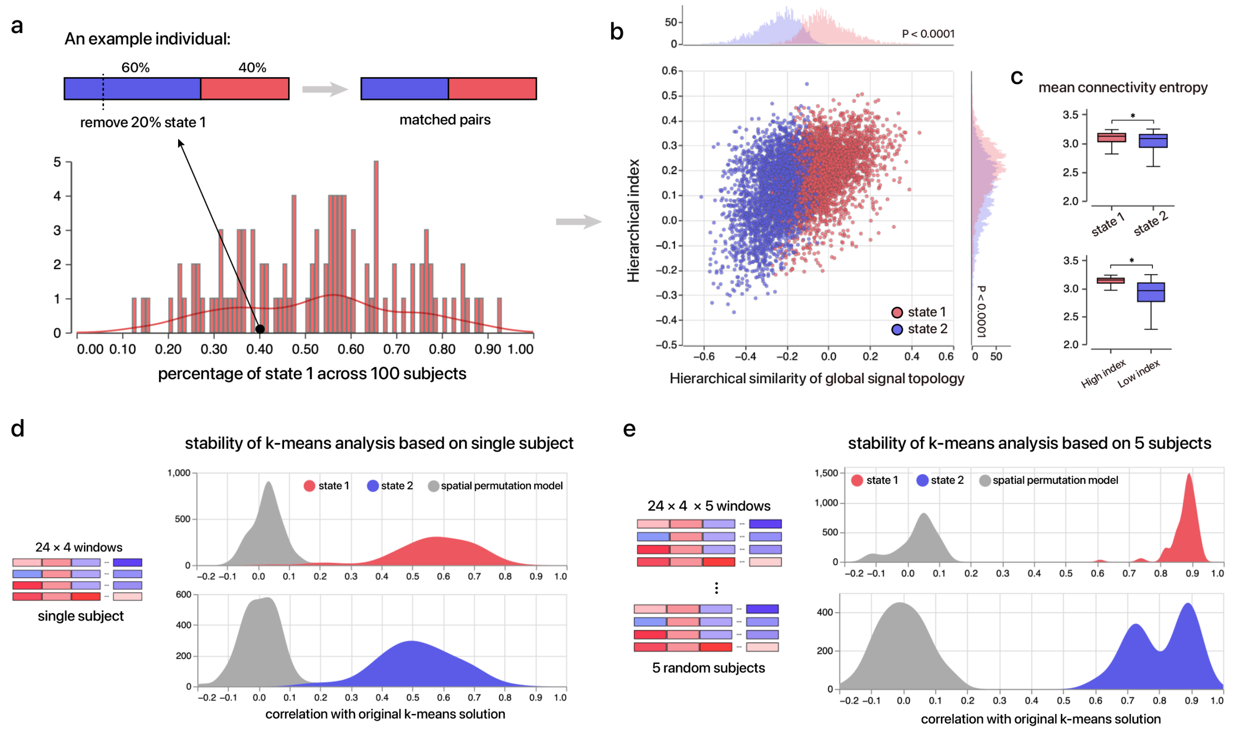

Fig S8. Supplementary analyses to evaluate the impact of inter-individual variations on the clustering of dynamic brain states. **a,** In 100 unrelated subjects, the K-means clustering method assigned a proportion of individuals to state 1, with each individual having 4×24 sample points; to mitigate the impact of inter-individual discrepancies, in subsequent analyses (**b-c**), we ensured that the sample points of the two states were balanced; for example, for a hypothetical sample, if state 1 represented 40% of the total, then we would eliminate 20% of state 2 to ensure that state 1 and state 2 were equally distributed. **b**, 2-cluster solution of the GS topology in 3840 balanced time windows from 100 unrelated HCP individuals. Scatter and distribution plots of the hierarchical index; the hierarchical similarity with the GS topology is shown. Each point represents a 35 s fragment. State 1 has significantly larger hierarchical index ($P < .0001$, two-sided two-sample t-test) and hierarchical similarity with GS topology ($P < .0001$, two-sided two-sample t-test) than State 2, indicating a higher level of vigilance and more association regions contributing to global fluctuations; meanwhile, the two variables are moderately correlated ($r = 0.47$, $P < .0001$, two-sided unadjusted Spearman correlation). **c**, Top: Higher overall connectivity entropy in State 1 than State 2 ($P = 6.2\times10^{-21}$, $n_{state\ 1} = 3285$, $n_{state\ 2} = 3285$, two-sided two-sample t-test). Bottom: Higher overall connectivity entropy in states with a higher hierarchical index (top 20% versus bottom 20%; $P < .0001$, two-sided two-sample t-test, $n_{high} = 1920$, $n_{low} = 1920$). In each box plot, the midline represents the median, and its lower and upper edges represent the first and third quartiles, and whiskers represent the 1.5 × interquartile range. **d,** The stability of the 2-solution K-means analysis based on a single subject (96 runs) was assessed by 100 independent experiments. The upper right panel shows the spatial correlation between the averaged cluster center and the group-level state 1 center; the gray area indicates the correlation based on the group center generated by the spatially permuted null model; the lower right panel corresponds to state 2. The highest correlation was chosen to match individual and group states in each independent experiment. **e,** The stability of the 2-solution K-means analysis based on five randomly selected subjects (65 × 5 runs) was assessed by 100 independent experiments. Other parts are consistent with d.

Supplementary Figure 9

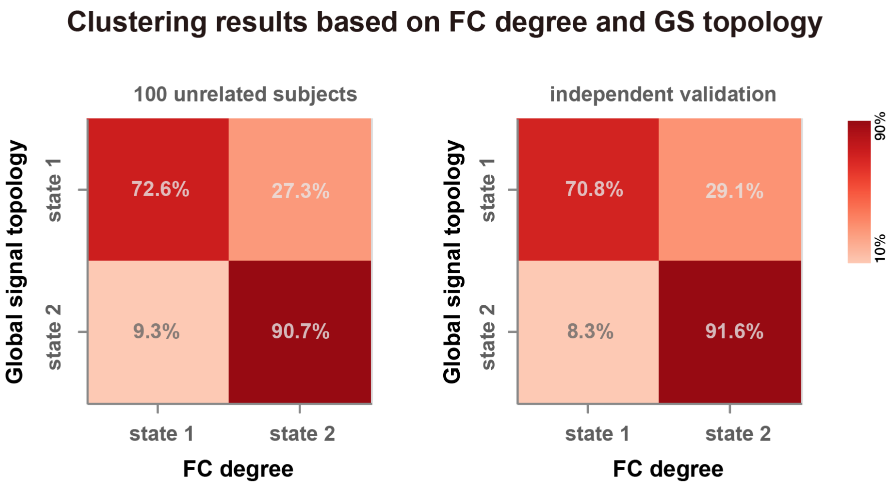

Fig S9. Comparison of 2-cluster solution of 9,600 time windows using GS topology and FC degree patterns. Diagonal elements represent the percentage of two approaches that achieved the same clustering result.

Supplementary Figure 10

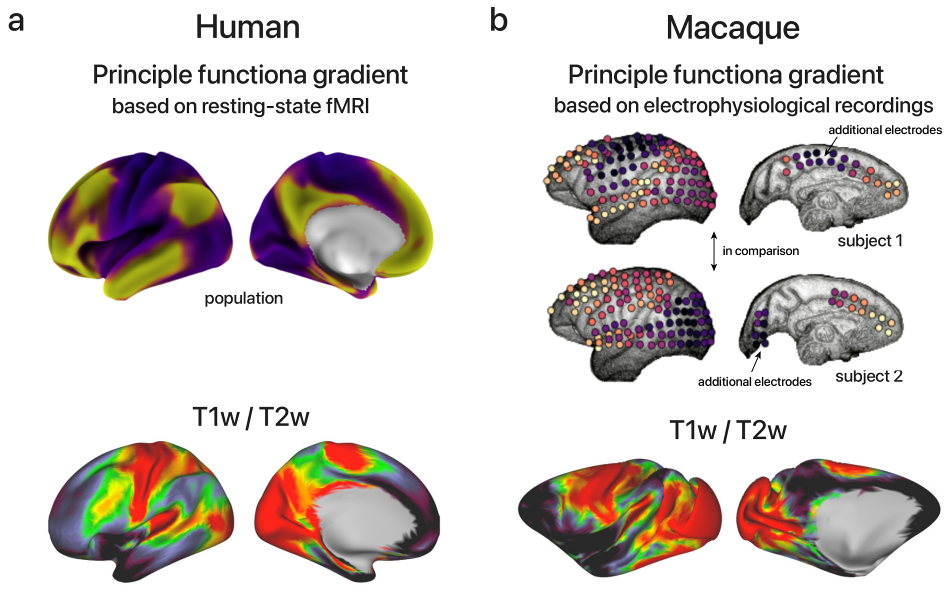

Fig S10. Human and macaque cortical hierarchical organization. **a**, Top: principal gradient of the functional connectome based on human resting-state fMRI data. Bottom: human cortical T1w/T2w contrast. **b**, Top: principal gradient of the functional connectome based on resting-state macaque ECoG data; two macaques. Bottom: macaque cortical T1w/T2w contrast. The T1w/T2w ratio maps shown here were from a previous study[6].

Supplementary Figure 11

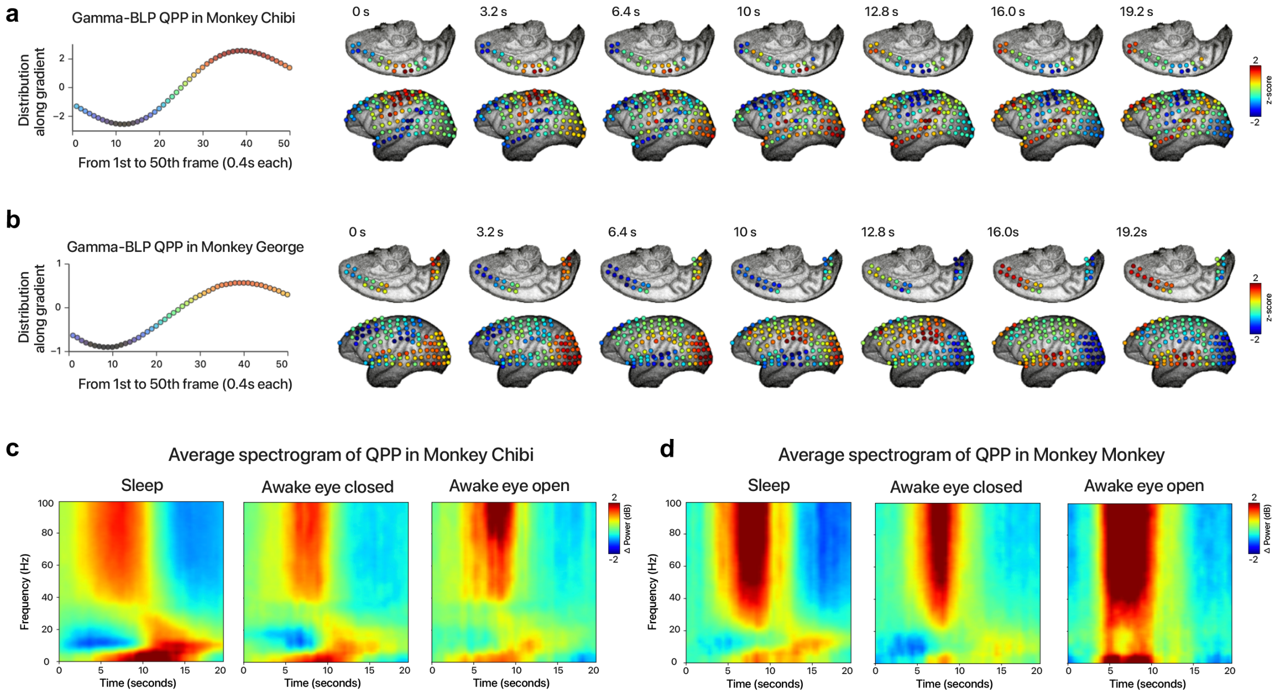

Fig S11. QPP analysis of spatiotemporal gamma-BLP dynamics based on macaque electrocorticography recordings. **a-b,** Recurrent spatiotemporal dynamics in Macaque *Chibi* (a; same in Fig 6a-b) and Macaque *George* (b). Left: typical cycle of gamma-BLP QPP; x-axis: HCP temporal frames (0.4 second each), y-axis: dot product of gamma-BLP values and principal functional gradient. Right**:** representative frames across 20 seconds. To better visualize the spatial topography, the mean value was subtracted in each frame across the typical gamma-BLP QPP template. **b,** Recurrent spatiotemporal dynamics in Macaque *George*, see above. **c-d,** Average spectrogram of QPP events detected in Macaque *Chibi* (c) and Macaque *George* (d) across sleep, awake eyes-closed, as well as awake eyes-opened, recording sessions. The results are highly analogous to sequential spectral transition (SST) events.

Supplementary Figure 12

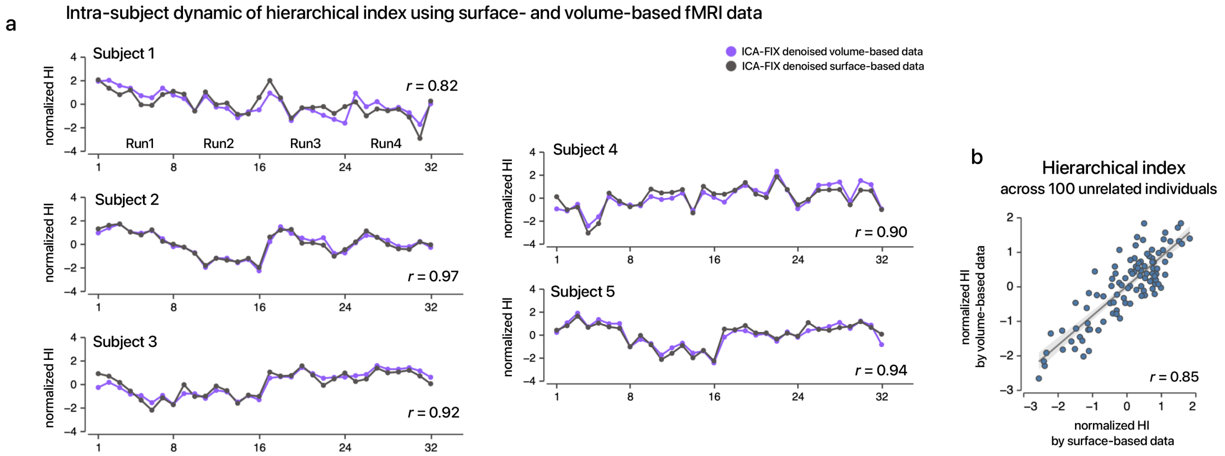

Fig. S12. a, Intra-subject correlation: the dynamic trajectory of the hierarchical index based on surface- and volume-based ICA-FIX-denoised resting-state fMRI data over four 15-min resting state runs in five subjects (each point represents 150 TRs, up to 32 non-overlapping windows). **b**, Inter-subject correlation: the inter-individual difference in the hierarchical index based on surface- and volume-based ICA-FIX-denoised resting-state fMRI data from 100 unrelated subjects. The hierarchical index was averaged across four runs for each subject. The error band represents the 95% confidence interval of the regression estimate.

Supplementary Figure 13

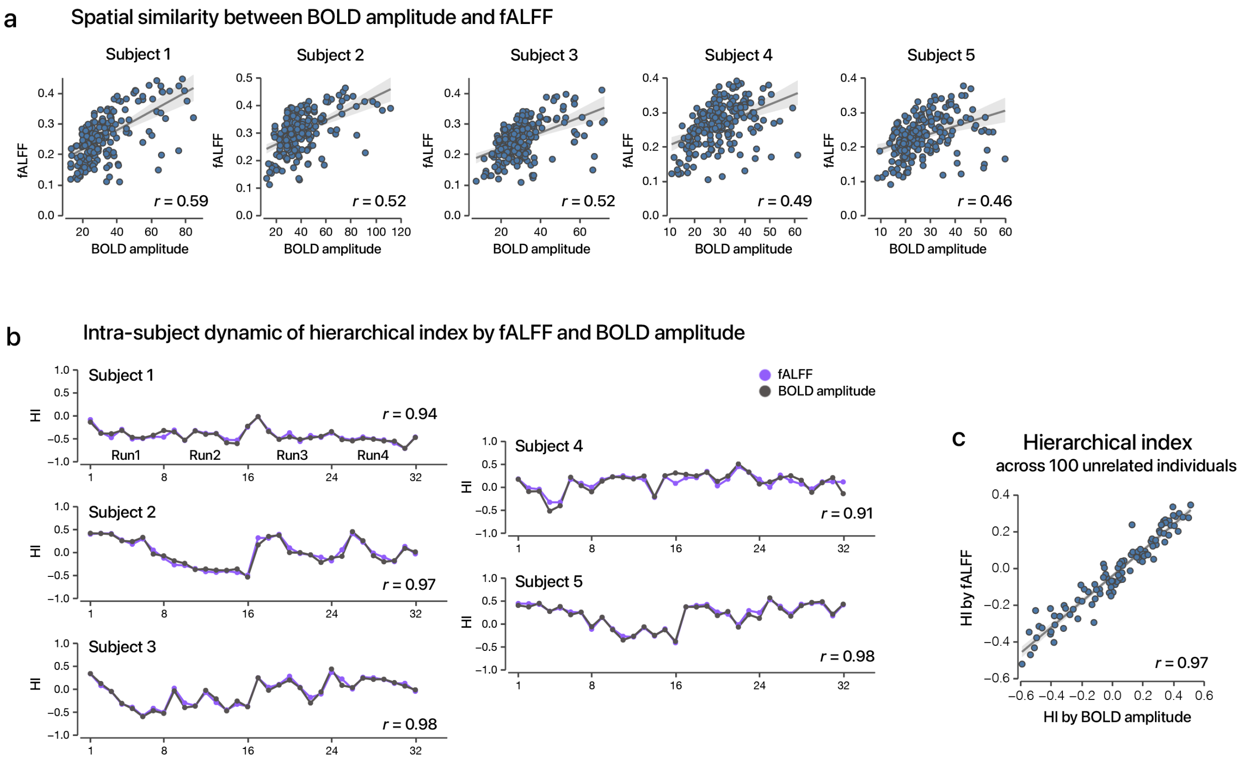

Fig S13. BOLD variability/amplitude and fractional amplitude of low-frequency fluctuation (fALFF)**. a,** Spatial similarity: BOLD variability and fALFF maps showed significant spatial similarity in 5 unrelated subjects randomly selected from HCP data (each point represents a region in Schaefer atlas with 200 parcellation). The error band represents the 95% confidence interval of the regression estimate. **b,** Intra-subject correlation: the dynamic flow of hierarchical index based on BOLD variability or fALFF measurement across four 15 min resting state runs in above five subjects (each point represents 150 TRs). **c,** Inter-subject correlation: the inter-individual difference in hierarchical index based on BOLD variability or fALFF across 15 min resting-state scanning from 100 unrelated subjects. The error band represents the 95% confidence interval of the regression estimate.

Supplementary Figure 14

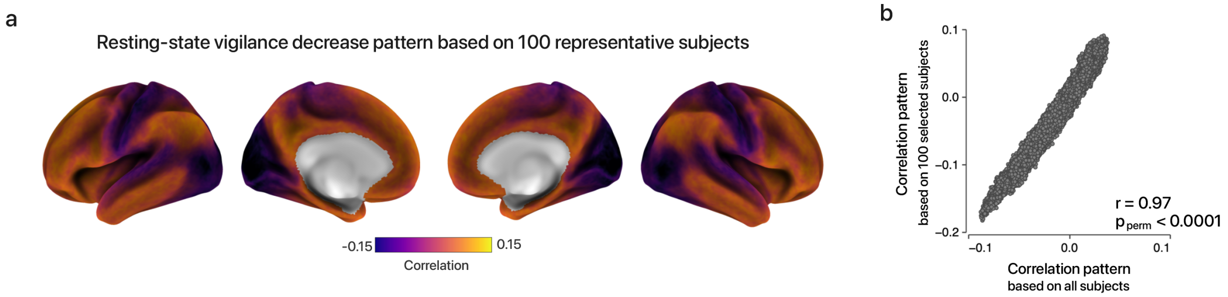

Fig. S14. Vigilance decrease pattern based on 100 representative subjects (greatest degree of reduction in hierarchical index from time trend analysis). **a**, Cortex-wide unthresholded correlation map between time intervals and z-normalized BOLD amplitude; a negative correlation indicates that the amplitude became more larger along with scanning time and vice versa. **b**, Vigilance-related cortical maps are highly similar across the spatial distribution using all or 100 selected subjects. (r = 0.97, P_{perm} < .0001, Spearman rank correlation).

Supplementary Figure 15

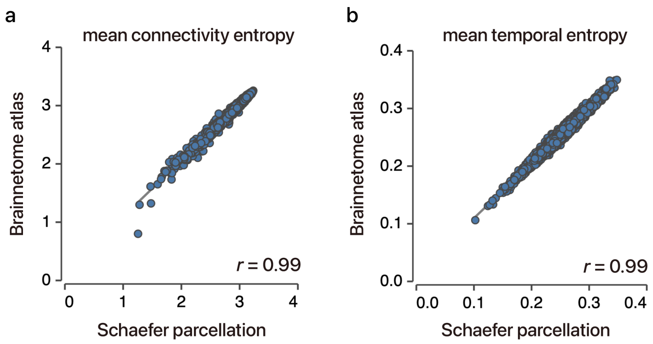

Fig. S15. The entropy measurements based on ROIs defined by the Brainnetome Atlas and Schaefer's parcellation are highly consistent across 9,600 brain states (100 subjects × 24 windows × 4 runs). **a**, connectivity entropy. **b**, temporal entropy.

Supplementary Figure 16

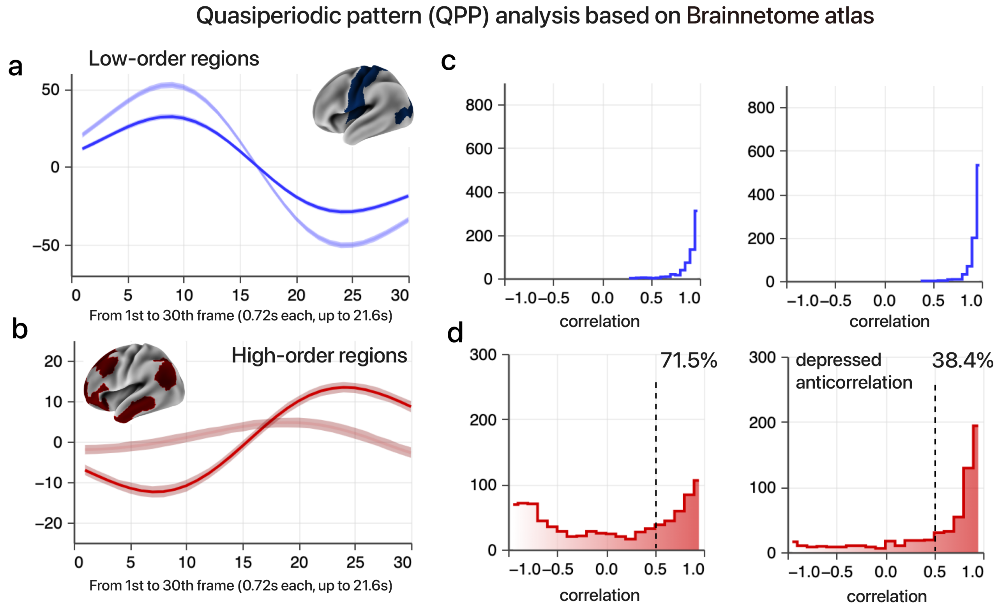

Fig. S16. a-b, Group-averaged QPP events detected in different vigilance states (initial and terminal 400 frames, respectively) based on QPP anlaysis. For this visualization, the time series of the bottom 20% (a, blue) and top 20% (b, red) of the hierarchy regions were averaged across 30 frames. Greater saturability corresponds to the initial 400 frames with plausibly higher vigilance. The error band indicates the 95% confidence interval. **c-d,** Distribution of the temporal correlations between the averaged time series in the template and all the detected QPP events. Left: Higher vigilance; right: lower vigilance. For the top 20% multimodal areas, an r threshold of 0.5 was displayed to highlight the heterogeneity between the two states.

Supplementary Figure 17

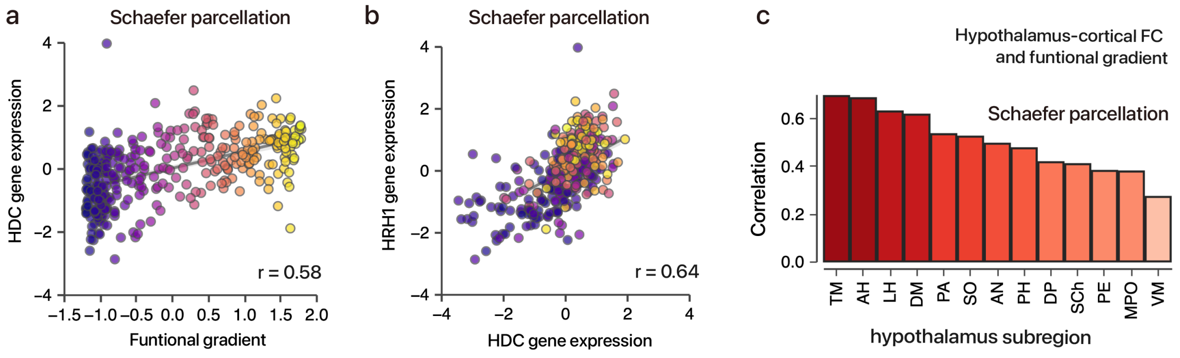

Figure S17. **a-b**, Gene expression pattern of the HDC is highly correlated with functional hierarchy (r = 0.58) and the expression of the HRH1 gene (r = 0.64). The error band represents the 95% confidence interval of the regression estimate. **c**, Spatial association between hypothalamic subregions functional connection to cortical area and functional gradient. The tuberomammillary nucleus showed one of the most outstanding correlations. From left to right: tuberomammillary nucleus (TM), anterior hypothalamic area (AH), dorsomedial hypothalamic nucleus (DM), lateral hypothalamus (LH), paraventricular nucleus (PA), arcuate nucleus (AN), suprachiasmatic nucleus (SCh), dorsal periventricular nucleus (DP), medial preoptic nucleus (MPO), periventricular nucleus (PE), posterior hypothalamus (PH), ventromedial nucleus (VM).

Supplementary Figure 18

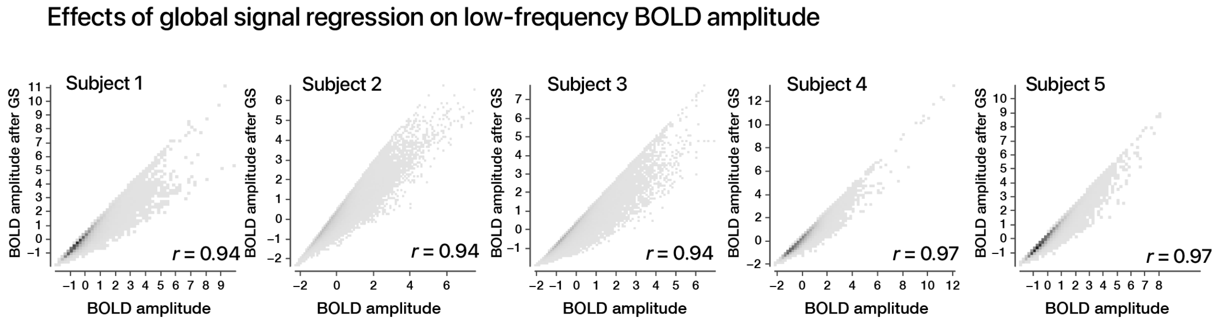

Fig S18. Global signal regression step has minimal effect on the spatial organization of BOLD amplitude across the cortex.

Supplementary Table 1. Two-sided Partial correlation results between averaged hierarchical index and behavioural measurements (unadjusted P value was shown).

HCP phenotype	Sample size	Partial correlation	CI 95%	Uncorrected P	Power
PSQI_AmtSleep	982	0.1939	[0.13 0.25]	<0.000001	1.0000
PSQI_Comp3	982	-0.1671	[-0.23 -0.11]	<0.000001	0.9996
DDisc_SV_1yr_200	981	0.1453	[0.08 0.21]	0.000005	0.9956
DDisc_AUC_40K	981	0.1436	[0.08 0.2]	0.000006	0.9948
Emotion_Task_Shape_Median_RT	977	-0.1412	[-0.2 -0.08]	0.000009	0.9934
WM_Task_Acc	982	0.1394	[0.08 0.2]	0.000012	0.9925
PicVocab_AgeAdj	982	0.1375	[0.08 0.2]	0.000015	0.9912
Language_Task_Median_RT	980	-0.1367	[-0.2 -0.07]	0.000018	0.9904
DDisc_SV_10yr_40K	981	0.1357	[0.07 0.2]	0.000020	0.9897
DDisc_SV_5yr_40K	981	0.1346	[0.07 0.2]	0.000024	0.9886
CogCrystalComp_AgeAdj	977	0.1342	[0.07 0.2]	0.000026	0.9879
Language_Task_Story_Median_RT	980	-0.1333	[-0.19 -0.07]	0.000028	0.9873
DDisc_AUC_200	981	0.1303	[0.07 0.19]	0.000042	0.9839
DDisc_SV_3yr_40K	981	0.1301	[0.07 0.19]	0.000044	0.9835
PicVocab_Unadj	982	0.1297	[0.07 0.19]	0.000045	0.9832
CogTotalComp_AgeAdj	970	0.1291	[0.07 0.19]	0.000055	0.9813
WM_Task_0bk_Tool_Acc_Nontarget	974	0.1289	[0.07 0.19]	0.000055	0.9812
WM_Task_2bk_Acc	974	0.1287	[0.07 0.19]	0.000057	0.9809
DDisc_SV_10yr_200	981	0.1279	[0.07 0.19]	0.000059	0.9804
Emotion_Task_Median_RT	977	-0.1272	[-0.19 -0.07]	0.000067	0.9790
CogCrystalComp_Unadj	977	0.1246	[0.06 0.19]	0.000094	0.9745
Language_Task_Story_Avg_Difficulty_Level	980	0.1229	[0.06 0.18]	0.000115	0.9714
Relational_Task_Acc	972	0.1217	[0.06 0.18]	0.000143	0.9677
Social_Task_Median_RT_TOM	977	-0.1197	[-0.18 -0.06]	0.000176	0.9638
CogTotalComp_Unadj	970	0.1173	[0.05 0.18]	0.000252	0.9559
DDisc_SV_6mo_200	981	0.1163	[0.05 0.18]	0.000261	0.9551
WM_Task_0bk_Tool_Acc	974	0.1167	[0.05 0.18]	0.000263	0.9549
ReadEng_AgeAdj	982	0.1150	[0.05 0.18]	0.000304	0.9513
WM_Task_0bk_Face_Median_RT	974	-0.1152	[-0.18 -0.05]	0.000317	0.9501
WM_Task_2bk_Face_Acc	974	0.1150	[0.05 0.18]	0.000322	0.9496
Relational_Task_Rel_Acc	972	0.1140	[0.05 0.18]	0.000369	0.9459
DDisc_SV_1yr_40K	981	0.1134	[0.05 0.17]	0.000374	0.9454
DDisc_SV_3yr_200	981	0.1130	[0.05 0.17]	0.000390	0.9443
WM_Task_0bk_Median_RT	971	-0.1129	[-0.17 -0.05]	0.000425	0.9416

Social_Task_TOM_Median_RT_TOM	979	-0.1120	[-0.17 -0.05]	0.000449	0.9399
WM_Task_0bk_Place_Acc_Nontarget	974	0.1122	[0.05 0.17]	0.000450	0.9399
WM_Task_0bk_Face_Median_RT_Nontarget	974	-0.1121	[-0.17 -0.05]	0.000456	0.9394
ReadEng_Unadj	982	0.1106	[0.05 0.17]	0.000518	0.9352
WM_Task_2bk_Place_Acc	974	0.1063	[0.04 0.17]	0.000894	0.9140
WM_Task_0bk_Tool_Median_RT_Nontarget	974	-0.1049	[-0.17 -0.04]	0.001043	0.9070
WM_Task_0bk_Acc	982	0.1034	[0.04 0.16]	0.001178	0.9011
WM_Task_0bk_Tool_Median_RT	974	-0.1024	[-0.16 -0.04]	0.001377	0.8931
Emotion_Task_Face_Median_RT	977	-0.1017	[-0.16 -0.04]	0.001464	0.8897
DDisc_SV_6mo_40K	981	0.1009	[0.04 0.16]	0.001549	0.8866
Emotion_Task_Shape_Acc	977	0.1011	[0.04 0.16]	0.001551	0.8866
WM_Task_2bk_Body_Acc_Target	974	0.0975	[0.03 0.16]	0.002322	0.8619
WM_Task_2bk_Body_Acc	974	0.0972	[0.03 0.16]	0.002390	0.8599
WM_Task_2bk_Face_Acc_Target	974	0.0967	[0.03 0.16]	0.002513	0.8565
Language_Task_Acc	980	0.0947	[0.03 0.16]	0.003003	0.8438
DDisc_SV_5yr_200	981	0.0939	[0.03 0.16]	0.003256	0.8377
Social_Task_TOM_Perc_TOM	980	0.0921	[0.03 0.15]	0.003889	0.8237
ProcSpeed_Unadj	982	0.0911	[0.03 0.15]	0.004295	0.8155
ProcSpeed_AgeAdj	982	0.0909	[0.03 0.15]	0.004352	0.8144
Language_Task_Math_Median_RT	980	-0.0901	[-0.15 -0.03]	0.004769	0.8064
WM_Task_2bk_Face_Median_RT_Target	967	-0.0899	[-0.15 -0.03]	0.005156	0.7994
Language_Task_Story_Acc	980	0.0889	[0.03 0.15]	0.005328	0.7964
WM_Task_0bk_Place_Median_RT	974	-0.0889	[-0.15 -0.03]	0.005491	0.7937
WM_Task_0bk_Place_Median_RT_Nontarget	974	-0.0872	[-0.15 -0.02]	0.006494	0.7776
WM_Task_2bk_Face_Acc_Nontarget	974	0.0870	[0.02 0.15]	0.006589	0.7761
WM_Task_0bk_Body_Median_RT	974	-0.0865	[-0.15 -0.02]	0.006880	0.7718
Relational_Task_Match_Acc	972	0.0863	[0.02 0.15]	0.007092	0.7688
WM_Task_Median_RT	969	-0.0860	[-0.15 -0.02]	0.007382	0.7647
WM_Task_0bk_Place_Acc	974	0.0857	[0.02 0.15]	0.007436	0.7639
WM_Task_2bk_Place_Acc_Nontarget	974	0.0853	[0.02 0.15]	0.007728	0.7599
CogEarlyComp_AgeAdj	974	0.0845	[0.02 0.15]	0.008307	0.7522
Emotion_Task_Acc	977	0.0841	[0.02 0.15]	0.008559	0.7490
CogFluidComp_AgeAdj	971	0.0842	[0.02 0.15]	0.008648	0.7479
PSQI_Score	982	-0.0829	[-0.14 -0.02]	0.009320	0.7396
WM_Task_0bk_Body_Median_RT_Nontarget	974	-0.0830	[-0.15 -0.02]	0.00952	0.7372
WM_Task_0bk_Body_Median_RT_Target	938	-0.0843	[-0.15 -0.02]	0.00977	0.7343

Supplementary Table 2. Top 100 genes whose expression pattern showing high spatial correlation with principal functional gradient across the cortex (two-sided unadjusted Spearman correlation).

gene_symbol	gene_name	Correlation	P value
**CAPN12**	calpain 12	0.7509442014296380	4.32327106001372E-20
**NT5DC2**	5'-nucleotidase domain containing 2	0.722415448629041	4.90044313578215E-18
**HDC**	histidine decarboxylase	0.716312813400192	1.24979671535625E-17
**CDH8**	cadherin 8, type 2	0.7034353995519050	8.33450286628482E-17
**SHC1**	SHC (Src homology 2 domain containing) transforming protein 1	0.694196095166969	3.05811623686686E-16
**NEK2**	NIMA (never in mitosis gene a)-related kinase 2	0.6833351114904510	1.32614671209208E-15
**CARTPT**	CART prepropeptide	0.6679291582204200	9.56378027943481E-15
**FAM110C**	family with sequence similarity 110, member C	0.6655393150538780	1.28586440989599E-14
**EHBP1L1**	EH domain binding protein 1-like 1	0.664547103382055	1.45287339087666E-14
**COL23A1**	collagen, type XXIII, alpha 1	0.6620078950176040	1.98168141896667E-14
**TNN**	tenascin N	0.6600234716739570	2.52047981825872E-14
**GPR120**	omega-3 fatty acid receptor 1	0.6589672463458870	2.8625790192358E-14
**NLE1**	notchless homolog 1 (Drosophila)	0.6568334578043320	3.69616597733905E-14
**PRTN3**	proteinase 3	0.6566947615491310	3.75781319863389E-14
**SPRN**	shadow of prion protein homolog (zebrafish)	0.6549557238877630	4.62051512001017E-14
**SCARA5**	scavenger receptor class A, member 5 (putative)	0.6534193961378430	5.53983683397363E-14
**BAIAP3**	BAI1-associated protein 3	0.6524805291795580	6.1863384448077E-14
**KCNB2**	potassium voltage-gated channel, Shab-related subfamily, member 2	0.6511362423983780	7.24065775544207E-14
**CIB1**	calcium and integrin binding 1 (calmyrin)	0.6509975461431770	7.35884946570596E-14
**TMEM108**	transmembrane protein 108	0.6505281126640350	7.77290251876036E-14
**ST6GALNAC5**	ST6 (alpha-N-acetyl-neuraminyl-2,3-beta-galactosyl-1,3)-N-acetylgalactosaminide alpha-2,6-sialyltransferase 5	0.6478502080443830	1.06024826553402E-13
**FAM148C**	C2 calcium-dependent domain containing 4C	0.6476368291902270	1.08665819527456E-13
**GULP1**	GULP, engulfment adaptor PTB domain containing 1	0.6467726448308970	1.20027967337897E-13
**GSG1**	germ cell associated 1	0.6462818734663400	1.26983806273185E-13
**TFPT**	TCF3 (E2A) fusion partner (in childhood Leukemia)	0.6460044809559370	1.31086281829353E-13
**KRT83**	keratin 83	0.6458871225861520	1.32860294394363E-13
**AC024270.1**	hepatoma-derived growth factor, related protein 3	0.6448842419716210	1.48997897956145E-13
**PCDH19**	protocadherin 19	0.6446601941747570	1.52854114098103E-13
**PHACTR3**	phosphatase and actin regulator 3	0.6446495252320500	1.53040132015134E-13
**SLC29A3**	solute carrier family 29 (nucleoside transporters), member 3	0.6424517230342470	1.9641415467632E-13
**FOXRED2**	FAD-dependent oxidoreductase domain containing 2	0.6416942281019950	2.13953860273951E-13
**PLCH2**	phospholipase C, eta 2	0.640648671716633	2.4067141895865E-13
**NAT14**	N-acetyltransferase 14 (GCN5-related, putative)	0.6396777979302250	2.68351195273915E-13
**STX1A**	syntaxin 1A (brain)	0.6391336818521290	2.85185331038125E-13
**ARHGAP28**	Rho GTPase activating protein 28	0.6373306305345140	3.48588740907342E-13

**CPNE8**	copine VIII	0.6371492585084820	3.55673779310313E-13
**DENND1C**	DENN/MADD domain containing 1C	0.6349834631388030	4.51809202751806E-13
**NOL4**	nucleolar protein 4	0.634407340232583	4.8134545188864E-13
**NOV**	nephroblastoma overexpressed	0.6279739677797930	9.67603166728629E-13
**SLC27A3**	solute carrier family 27 (fatty acid transporter), member 3	0.6265976741704900	1.12114550722948E-12
**B3GAT1**	beta-1,3-glucuronyltransferase 1 (glucuronosyltransferase P)	0.626202923290302	1.16936537475554E-12
**COL10A1**	collagen, type X, alpha 1	0.6255521177851280	1.25327015697498E-12
**C21orf56**	chromosome 21 open reading frame 56	0.6246559265976740	1.37838857334703E-12
**HSD11B1L**	hydroxysteroid (11-beta) dehydrogenase 1-like	0.6233756534727410	1.57826874687384E-12
**NR4A3**	nuclear receptor subfamily 4, group A, member 3	0.6231622746185850	1.61419646243171E-12
**TLN2**	talin 2	0.6228635442227680	1.66582548667151E-12
**ABR**	active BCR-related	0.6228208684519370	1.67333006773761E-12
**PHF20L1**	PHD finger protein 20-like 1	0.6214018990718020	1.942319099215E-12
**KIF21B**	kinesin family member 21B	0.6213805611863870	1.946666719777E-12
**HDAC9**	histone deacetylase 9	0.6187239944521500	2.56809141370922E-12
**VAV1**	vav 1 guanine nucleotide exchange factor	0.6184466019417480	2.6430696023526E-12
**CTXN1**	cortexin 1	0.6164728475408090	3.24103663244585E-12
**CITED4**	Cbp/p300-interacting transactivator, with Glu/Asp-rich carboxy-terminal domain, 4	0.6149365197908890	3.79491955363774E-12
**DPYSL4**	dihydropyrimidinase-like 4	0.6147124719940260	3.88296854123673E-12
**MYBPC1**	myosin binding protein C, slow type	0.6138482876346960	4.24143107916477E-12
**LOC100287347**	similar to hCG1777462	0.6132935026138910	4.48817498318443E-12
**KCNG1**	potassium voltage-gated channel, subfamily G, member 1	0.6129414275045340	4.65188972968254E-12
**KIAA1644**	KIAA1644	0.6114904512962770	5.38953249390832E-12
**NEURL1B**	neuralized homolog 1B (Drosophila)	0.6107863010775630	5.78701649554161E-12
**MPV17L2**	MPV17 mitochondrial membrane protein-like 2	0.6106155979942390	5.88755556411482E-12
**PDE4C**	phosphodiesterase 4C, cAMP-specific	0.610412888082791	6.00913636985068E-12
**CMTM8**	CKLF-like MARVEL transmembrane domain containing 8	0.6093886695828450	6.66144095397341E-12
**SNX7**	sorting nexin 7	0.6093566627547210	6.68288866946789E-12
**LASS1**	ceramide synthase 1	0.60921796649952	6.77660139888367E-12
**FILIP1L**	filamin A interacting protein 1-like	0.6092072975568120	6.7838623684407E-12
**WDR23**	DDB1 and CUL4 associated factor 11	0.6085564920516380	7.24126670356625E-12
**L3MBTL4**	l(3)mbt-like 4 (Drosophila)	0.6080017070308330	7.65456018385678E-12
**MUM1L1**	melanoma associated antigen (mutated) 1-like 1	0.6077563213485550	7.84453993939487E-12
**ASS1**	argininosuccinate synthase 1	0.6071161847860880	8.36180359794138E-12
**SH2D5**	SH2 domain containing 5	0.6069561506454710	8.49617413693701E-12
**VASN**	vasorin	0.6064653792809130	8.92136287331538E-12
**GSS**	glutathione synthetase	0.6063906966819590	8.98783925948294E-12
**MARCH1**	membrane-associated ring finger (C3HC4) 1, E3 ubiquitin protein ligase	0.6058359116611550	9.49684683467615E-12
**C1orf145**	chromosome 1 open reading frame 145	0.6055905259788760	9.73075454137598E-12

**ADAMTS3**	ADAM metallopeptidase with thrombospondin type 1 motif, 3	0.6054945054945060	9.82378852750879E-12
**DDRGK1**	DDRGK domain containing 1	0.6045022938226820	1.0836787858085E-11
**CCDC41**	coiled-coil domain containing 41	0.6041502187133260	1.12199007583169E-11
**RPL36**	ribosomal protein L36	0.6039155019737540	1.14825334779147E-11
**HECTD2**	HECT domain containing E3 ubiquitin protein ligase 2	0.603318041182119	1.2178092847373E-11
**KCNA5**	potassium voltage-gated channel, shaker-related subfamily, member 5	0.6032967032967030	1.22036712322931E-11
**NFKBIE**	nuclear factor of kappa light polypeptide gene enhancer in B-cells inhibitor, epsilon	0.6031686759842100	1.23582333500393E-11
**FIP1L1**	FIP1 like 1 (S. cerevisiae)	0.6026458977915290	1.3009189788455E-11
**GLRA3**	glycine receptor, alpha 3	0.6023578363384190	1.33818865830372E-11
**CAMK2D**	calcium/calmodulin-dependent protein kinase II delta	0.6015469966926280	1.44870917401841E-11
**LMF1**	lipase maturation factor 1	0.6014616451509660	1.46084235804416E-11
**CCDC68**	coiled-coil domain containing 68	0.6012802731249330	1.4869516596302E-11
**CYP46A1**	cytochrome P450, family 46, subfamily A, polypeptide 1	0.6009922116718240	1.5293468790088E-11
**FAM160A2**	family with sequence similarity 160, member A2	0.6006401365624670	1.58274796806859E-11
**C6orf222**	chromosome 6 open reading frame 222	0.6001386962552010	1.66191897572836E-11
**ANKRD55**	ankyrin repeat domain 55	0.6000853515416620	1.67056276696729E-11
**MED4**	mediator complex subunit 4	0.5991891603542090	1.82242764652544E-11
**LGI4**	leucine-rich repeat LGI family, member 4	0.5986663821615280	1.91707457756039E-11
**TMEM54**	transmembrane protein 54	0.5984423343646650	1.9590728872329E-11
**RAB36**	RAB36, member RAS oncogene family	0.5979942387709380	2.04574708448234E-11
**KLHDC8B**	kelch domain containing 8B	0.5978875493438600	2.06692238133875E-11
**C11orf95**	chromosome 11 open reading frame 95	0.5966819588178810	2.32137946421773E-11
**C2orf80**	chromosome 2 open reading frame 80	0.5966499519897580	2.32853044881091E-11
**AGRN**	agrin	0.5966179451616350	2.33570266977593E-11
**PALM**	paralemmin	0.5963618905366480	2.39385226070256E-11
**SCN3B**	sodium channel, voltage-gated, type III, beta subunit	0.5961058359116610	2.45339635996899E-11

Supplementary Table 3. The top 11 negatively correlated genes with the principal functional gradient, out of 100 genes with the strongest spatial association (based on absolute correlation value, (two-sided unadjusted Spearman correlation).

gene_symbol	gene_name	Correlation	P value
SHC1	SHC (Src homology 2 domain containing) transforming protein 1	-0.7011522	1.15E-16
NOL4	nucleolar protein 4	-0.6355382	4.25E-13
NR4A3	nuclear receptor subfamily 4, group A, member 3	-0.6247413	1.37E-12
ABR	active BCR-related	-0.6241972	1.45E-12
LOC100287347	similar to hCG1777462	-0.6171237	3.03E-12
PDE4C	phosphodiesterase 4C, cAMP-specific	-0.6137309	4.29E-12
FILIP1L	filamin A interacting protein 1-like	-0.6112344	5.53E-12
KCNA5	potassium voltage-gated channel, shaker-related subfamily, member 5	-0.6061666	9.19E-12
RAB36	RAB36, member RAS oncogene family	-0.6013123	1.48E-11
C2orf80	chromosome 2 open reading frame 80	-0.6006615	1.58E-11
SHC1	SHC (Src homology 2 domain containing) transforming protein 1	-0.7011522	1.15E-16

Supplementary Table 4. Summary of consciousness-related studies comparing measurements relevant to BOLD amplitude.

publication	Localized BOLD measurement	Paradigm	Species	Sample size	Relevant results
He et al, 2014[7]	ALFF (0.01-0.08 Hz)	Disorders of consciousness (DOC)	Human	24	Patients versus controls: increased 'activation' in the insula, lingual gyrus, paracentral and supplementary motor area; decreased 'activation' in midline areas including MPFC, ACC, PCC.
Guo et al, 2019[8]	ALFF	Severe Traumatic Brain Injury	Human	42	Patients with sTBI showed significantly decreased FC and ALFF values in the DMN. However, patients with a better prognosis showed a significant increase ALFF values in the DMN.
Tsai et al, 2013[9]	ALFF (0.01–0.1 Hz)	Stroke patients with impairment in consciousness	Human	34	The intensity of ALFF was significantly decreased in the precuneus and posterior cingulate cortex regions among stroke patients with impaired consciousness
Huang et al, 2014[10]	ALFF (0.01–0.10 Hz)	Disorders of consciousness (DOC)	Human	23	Lower ALFF in perigenual anterior cingulate cortex (PACC) and posterior cingulate cortex (PCC) in DOC patients.
Baria et al, 2021[11]	BOLD variability (0.01 to 0.08 Hz)	Isoflurane anesthesia	Sprague-Dawley rats	48	fMRI BOLD variability was significant higher during the awake state compared with isoflurane anesthesia. Inferred from on *Table 2,* **Association areas** have larger effect sizes than **sensory cortices**.
Liu et al, 2017[12]	fALFF (0.01–0.1 Hz)	Propofol anesthesia	Human	15	fractional amplitude of LFF (fALFF index) was reduced in comparison to wakeful baseline in the anterior frontal regions, temporal pole, hippocampus, parahippocampal gyrus, and amygdala. Such changes were absent in large areas of the motor, parietal, and sensory cortices.
Huang et al, 2014[13]	standard deviation of the BOLD signal (0.01–0.10 Hz)	Propofol anesthesia and inspiratory sevoflurane anesthesia	Human	12	Abnormal balance between medial and lateral cortical networks in anesthesia: **z-normalized** temporal variance reduced in cortical midline regions (key nodes of DMN) and increased widespread lateral regions (marked by auditory cortex)
Huang et al, 2016[14]	Standard deviation of the BOLD signal (0.01–0.08 Hz)	Propofol anesthesia and inspiratory sevoflurane anesthesia	Human	12	Based on Figure 1, the temporal variance decreased preferentially in high-order regions during unconsciousness.

Yan et al, 2009[15]	ALFF (0.01-0.08 Hz)	eyes-closed (EC), eyes-open (EO), and eyes-open with a fixation (EO-F) condition	Human	20	The PCC and the MPFC showed significantly decreased ALFF in the EC compared with EO/EO-F condition; a small part of the parahippocampal gyrus showed increased ALFF in the EC compared with EO-F condition.
Liang et al, 2014[16]	fALFF (0.01–0.08 Hz)	eyes-closed (EC) and eyes-open (EO) condition	Human	24	Prominent relationships between increased fALFF for EC and decreased fALFF for EO in sensorimotor regions.
Liu et al, 2013[17]	ALFF (0.01-0.08 Hz)	eyes-closed (EC) and eyes-open (EO) condition	Human	48	Significantly higher ALFF in areas including the bilateral MOG and orbital frontal cortex in the EO relative to the EC, and lower ALFF in regions including the motor network (e.g., the bilateral primary sensorimotor cortex, supplementary motor area and PCL), the auditory cortex and insula and thalamus etc.
Wei et al, 2018[18]	fALFF (0.01–0.1 Hz)	eyes-closed (EC) and eyes-open (EO) condition	Human	48	EO resting state was associated with decreased fALFF values mainly in the primary and secondary sensory cortical areas, the insula, and the thalamus.
Tagliazucchi et al, 2013[19]	Temporal variance	Sleep	Human	63	No difference (with respect to wakefulness) for N1 sleep; however, N2 sleep was marked by an increase in signal variance that affected all sensory regions (visual, auditory, and somatosensory), and N3 sleep was characterized by an increase restricted to the visual cortex.

Supplementary Table 5. Analysis based on Anderson et al, 2020[20]: The top 10 genes whose expression patterns positively explained the spatial pattern of the principal functional gradient. Anderson et al generated and released the gene expression values in 400 parcel functional Schaefer atlas at: https://github.com/HolmesLab/2020_NatComm_interneurons_cortical_function_schizophrenia. Different from *adagen*'s toolbox using MNI coordinates from non-linear registration, this work derived cortical tissue samples from Freesurfer derived cortical surfaces for each donor in Allen Human Brain Atlas. After transforming to a common fsLR32k space, the samples were matched to nearest surface vertex.

Gene Symbol	Name	Spearman's Rho	Rank
FREM3	FRAS1 related extracellular matrix 3	0.597	1
NEK2	NIMA (never in mitosis gene a)-related kinase 2	0.587	2
TWIST2	twist homolog 2 (Drosophila)	0.586	3
SPON2	spondin 2, extracellular matrix protein	0.583	4
**HDC**	**histidine decarboxylase**	**0.579**	**5**
MUM1L1	melanoma associated antigen (mutated) 1-like 1	0.578	6
BAIAP3	BAI1-associated protein 3	0.577	7
GSG1	germ cell associated 1	0.571	8
LOC646627	phospholipase inhibitor	0.566	9
PPEF1	protein phosphatase, EF-hand calcium binding domain 1	0.561	10

Supplementary Table 6. Analysis in the *NeuroVault* website: The top 10 genes whose expression patterns positively explained the spatial pattern of the principal functional gradient. Detailed results could be easily assessed from https://neurovault.org/images/24346/gene_expression?q=&mask=cortex and the methodology was described in a published poster[21]. Briefly, a linear model was fitted to see how similar the transcriptomic profiles to functional gradient across the cortex.

Gene Symbol	Name	Explained Variance	Rank
CARTPT	CART prepropeptide	32.61%	1
NEK2	NIMA (never in mitosis gene a)-related kinase 2	27.21%	2
BAIAP3	BAI1-associated protein 3	25.52%	3
FAM148C	C2 calcium-dependent domain containing 4C	25.32%	4
GSG1	germ cell associated 1	25.08%	5
TWIST2	twist homolog 2 (Drosophila)	25.03%	6
ARHGAP28	Rho GTPase activating protein 28	25.03%	7
SPON2	spondin 2, extracellular matrix protein	23.78%	8
**HDC**	**histidine decarboxylase**	**22.93%**	**9**
KCNG1	potassium voltage-gated channel, subfamily G, member 1	21.89%	10

Supplementary Table 7. The vital signs of participants in the three conditions.

	Wakefulness	Moderate sedation	Recovery
MAP (mmHg)	90±12	83±12$^{*,\#}$	77±9*
HR (bpm)	67±12	58±7*	58±8*
SPO_2%	99.9±0.3	99.0±1.0	99.1±0.8
RR (bpm)	15±2	15±2	15±2
$PaCO_2$ (mmHg)	38.4±2.5	44.1±2.3*	41.5±2.8*

MAP, mean arterial pressure; HR, heart rate; SPO2, pulse oxygen saturation; RR, breathing rate; PaCO2, carbon dioxide arterial blood partial pressure. One-way analyses of variance (ANOVAs) and post hoc Tukey's honestly significant difference tests were performed using SPSS software (version 18.0, SPSS, Chicago, IL, USA).

* Values significantly different from wakefulness.
# Values significantly different from recovery in Tukey Honestly Significant Difference tests ($P < 0.05$).

1. Xu, K., Liu, Y., Zhan, Y., Ren, J. & Jiang, T. BRANT: A Versatile and Extendable Resting-State fMRI Toolkit. *Front Neuroinform* **12**, 52 (2018).
2. Friston, K. J., Williams, S., Howard, R., Frackowiak, R. S. & Turner, R. Movement-related effects in fMRI time-series. *Magn Reson Med* **35**, 346–355 (1996).
3. Glover, G. H., Li, T. Q. & Ress, D. Image-based method for retrospective correction of physiological motion effects in fMRI: RETROICOR. *Magn. Reson. Med.* **44**, (2000).
4. Alexander-Bloch, A. F., Shou, H., Liu, S., Satterthwaite, T. D., Glahn, D. C., Shinohara, R. T., Vandekar, S. N. & Raznahan, A. On testing for spatial correspondence between maps of human brain structure and function. *Neuroimage* **178**, (2018).
5. Vázquez-Rodríguez, B., Suárez, L. E., Markello, R. D., Shafiei, G., Paquola, C., Hagmann, P., Van Den Heuvel, M. P., Bernhardt, B. C., Spreng, R. N. & Misic, B. Gradients of structure–function tethering across neocortex. *Proc. Natl. Acad. Sci. U. S. A.* **116**, (2019).
6. Mars, R. B., Sotiropoulos, S. N., Passingham, R. E., Sallet, J., Verhagen, L., Khrapitchev, A. A., Sibson, N. & Jbabdi, S. Whole brain comparative anatomy using connectivity blueprints. *Elife* **7**, (2018).
7. He, J. H., Yang, Y., Zhang, Y., Qiu, S. Y., Zhou, Z. Y., Dang, Y. Y., Dai, Y. W., Liu, Y. J. & Xu, R. X. Hyperactive external awareness against hypoactive internal awareness in disorders of consciousness using resting-state functional MRI: Highlighting the involvement of visuo-motor modulation. *NMR Biomed.* **27**, (2014).
8. Guo, H., Liu, R., Sun, Z., Liu, B., Xiang, Y., Mao, J., Li, G. & Zhang, M. Evaluation of Prognosis in Patients with Severe Traumatic Brain Injury Using Resting-State Functional Magnetic Resonance Imaging. *World Neurosurg.* **121**, (2019).
9. Tsai, Y. H., Yuan, R., Huang, Y. C., Yeh, M. Y., Lin, C. P. & Biswal, B. B. Disruption of brain connectivity in acute stroke patients with early impairment in consciousness. *Front. Psychol.* **4**, (2014).
10. Huang, Z., Dai, R., Wu, X., Yang, Z., Liu, D., Hu, J., Gao, L., Tang, W., Mao, Y., Jin, Y., Wu, X., Liu, B., Zhang, Y., Lu, L., Laureys, S., Weng, X. & Northoff, G. The self and its resting state in consciousness: An investigation of the vegetative state. *Hum. Brain Mapp.* **35**, (2014).
11. Baria, A. T., Centeno, M. V., Ghantous, M. E., Chang, P. C., Procissi, D. & Apkarian, A. V. Bold temporal variability differentiates wakefulness from anesthesia-induced unconsciousness. *J. Neurophysiol.* **119**, (2018).
12. Gross, W., Liu, S., Lauer, K. K., Rohloff, R., Xu, Z., Douglas Ward, B., Binder, J. R., Chen, G., Li, S.-J., Hudetz, A. G., Gollapudy, S., Roberts, C. & Liu, X. Propofol attenuates low-frequency fluctuations of resting-state fMRI BOLD signal in the anterior frontal cortex upon loss of consciousness. *Neuroimage* **147**, 295–301 (2016).
13. Huang, Z., Wang, Z., Zhang, J., Dai, R., Wu, J., Li, Y., Liang, W., Mao, Y., Yang, Z., Holland, G., Zhang, J. & Northoff, G. Altered temporal variance and neural synchronization of spontaneous brain activity in anesthesia. *Hum. Brain Mapp.* **35**, 5368–5378 (2014).
14. Huang, Z., Zhang, J., Wu, J., Qin, P., Wu, X., Wang, Z., Dai, R., Li, Y., Liang, W., Mao, Y., Yang, Z., Zhang, J., Wolff, A. & Northoff, G. Decoupled temporal variability and signal synchronization of spontaneous brain activity in loss of consciousness: An fMRI study in anesthesia. *Neuroimage* **124**, 693–703 (2016).
15. Yan, C., Liu, D., He, Y., Zou, Q., Zhu, C., Zuo, X., Long, X. & Zang, Y. Spontaneous brain activity in the default mode network is sensitive to different resting-state conditions with limited cognitive load. *PLoS One* **4**, (2009).
16. Liang, B., Zhang, D., Wen, X., Xu, P., Peng, X., Huang, X., Liu, M. & Huang, R. Brain spontaneous fluctuations in sensorimotor regions were directly related to eyes open and eyes closed: Evidences from a machine learning approach. *Front. Hum. Neurosci.* **8**, (2014).
17. Liu, D., Dong, Z., Zuo, X., Wang, J. & Zang, Y. Eyes-open/eyes-closed dataset sharing for reproducibility evaluation of resting state fMRI data analysis methods. *Neuroinformatics* **11**, (2013).
18. Wei, J., Chen, T., Li, C., Liu, G., Qiu, J. & Wei, D. Eyes-open and eyes-closed resting states with opposite brain activity in sensorimotor and occipital regions: Multidimensional evidences from

machine learning perspective. *Front. Hum. Neurosci.* **12**, (2018).
19. Tagliazucchi, E., Von Wegner, F., Morzelewski, A., Brodbeck, V., Jahnke, K. & Laufs, H. Breakdown of long-range temporal dependence in default mode and attention networks during deep sleep. *Proc. Natl. Acad. Sci. U. S. A.* **110**, 15419–15424 (2013).
20. Anderson, K. M., Collins, M. A., Chin, R., Ge, T., Rosenberg, M. D. & Holmes, A. J. Transcriptional and imaging-genetic association of cortical interneurons, brain function, and schizophrenia risk. *Nat. Commun.* **11**, (2020).
21. Krzysztof J Gorgolewski, Andrew S Fox, Luke Chang, Alexander Schäfer, Katrin Arélin, Inga Burmann, Julia Sacher, D. S. M. Tight fitting genes: finding relations between statistical maps and gene expression patterns. *F1000Posters* **5:1607 Pos**, (2014).
