# Supplementary material for: Hierarchical fluctuation shapes a dynamic flow linked to states of consciousness
Source: Nat Commun. 2023 Jun 5;14:3238. doi: 10.1038/s41467-023-38972-x (PMC10241811; doi:10.1038/s41467-023-38972-x)
Supplement: Supplementary file 3 — Reporting Summary [file 41467_2023_38972_MOESM3_ESM.pdf]

nature portfolio

Corresponding author(s): Bing Liu, Xiaoqun Wang, Ruquan Han, Ang Li

Last updated by author(s): Apr 29, 2023

Reporting Summary

Nature Portfolio wishes to improve the reproducibility of the work that we publish. This form provides structure for consistency and transparency in reporting. For further information on Nature Portfolio policies, see our Editorial Policies and the Editorial Policy Checklist.

Statistics

For all statistical analyses, confirm that the following items are present in the figure legend, table legend, main text, or Methods section.

n/a	Confirmed	
☐	☒	The exact sample size (n) for each experimental group/condition, given as a discrete number and unit of measurement
☐	☒	A statement on whether measurements were taken from distinct samples or whether the same sample was measured repeatedly
☐	☒	The statistical test(s) used AND whether they are one- or two-sided<br>*Only common tests should be described solely by name; describe more complex techniques in the Methods section.*
☐	☒	A description of all covariates tested
☐	☒	A description of any assumptions or corrections, such as tests of normality and adjustment for multiple comparisons
☐	☒	A full description of the statistical parameters including central tendency (e.g. means) or other basic estimates (e.g. regression coefficient) AND variation (e.g. standard deviation) or associated estimates of uncertainty (e.g. confidence intervals)
☐	☒	For null hypothesis testing, the test statistic (e.g. F, t, r) with confidence intervals, effect sizes, degrees of freedom and P value noted<br>*Give P values as exact values whenever suitable.*
☒	☐	For Bayesian analysis, information on the choice of priors and Markov chain Monte Carlo settings
☒	☐	For hierarchical and complex designs, identification of the appropriate level for tests and full reporting of outcomes
☐	☒	Estimates of effect sizes (e.g. Cohen's d, Pearson's r), indicating how they were calculated

Our web collection on statistics for biologists contains articles on many of the points above.

Software and code

Policy information about availability of computer code

Data collection	For the dexmedetomidine-sedation experiment, fMRI data were collected using a Siemens Medical Systems Prisma 3.0 T MRI system at Beijing Neurosurgical Institute.For the sleeping experiment, the fMRI data were acquired using a 3T Siemens Trio scanner at the Sleep and Neuroimaging Center at Southwest University; simultaneously, the EEG recordings were conducted by a 32-channel MRI-compatible Brain Products system (BrainAmp MR plus, Brain products, Munich, Germany). To ensure the temporal stability of the EEG acquisition in relation to the switching of the gradients during the MR acquisition, we used a SyncBox (SyncBox MainUnit, Brain Products GmbH, Gilching, Germany) to synchronize the amplifier system with the MRI scanner's system. Please note: SyncBox is a hardware device and therefore providing a software version is not applicable.Other datasets used in this study are pre-existing datasets.
Data analysis	The original EEG recording was processed using the Vision-Analyzer software (version 2.0, Brain Products, Inc., Munich, Germany). The BrainSpace toolbox (version 0.1.1) was applied to calculate the principal gradient. The BRANT toolbox (version 3.35) for fMRI data preprocessing is available at https://github.com/kbxu/brant. The human gene expression was analyzed with abagen toolbox (version 0.0.8, https://abagen.readthedocs.io/en/stable/). The documentation and code for generating spatially-constrained null models are available at: https://github.com/netneurolab/markello_spatialnulls. QPP analysis was performed with code available here: https://github.com/FCP-INDI/C-PAC/blob/main/CPAC/qpp/qpp.py. The code used to compute the Shannon and Sample Entropy is available in the package NeuroKit2 (version 0.1.1, https://neuropsychology.github.io/NeuroKit/). The K-means clustering algorithm implemented in our study is based on the scikit-learn library (version 1.1.1, https://scikit-learn.org/stable/modules/clustering.html#k-means). Code to calculate hierarchical index at both volumetric and surface space in our work is available at Zenodo repository: https://doi.org/10.5281/zenodo.7855130.

For manuscripts utilizing custom algorithms or software that are central to the research but not yet described in published literature, software must be made available to editors and reviewers. We strongly encourage code deposition in a community repository (e.g. GitHub). See the Nature Portfolio guidelines for submitting code & software for further information.

Data

Policy information about availability of data

All manuscripts must include a data availability statement. This statement should provide the following information, where applicable:

- Accession codes, unique identifiers, or web links for publicly available datasets
- A description of any restrictions on data availability
- For clinical datasets or third party data, please ensure that the statement adheres to our policy

The raw and processed 3T and 7T resting-state fMRI, and group-average dense functional connectivity data are available from the Human Connectome Project (https://www.humanconnectome.org/study/hcp-young-adult/document/1200-subjects-data-release). Simultaneous EEG-fMRI validation data during sleep, collected by Gu et al89, can be downloaded at https://openneuro.org/datasets/ds003768/versions/1.0.9. The preprocessed MyConnectome Project data, originally analyzed here36, is publicly available at https://openneuro.org/datasets/ds000031/versions/00001. The preprocessed fMRI data during placebo and LSD conditions, first reported by Carhart-Harris et al40, can be accessed at https: //openneuro.org/datasets/ds003059. The resting-state fMRI of the Consortium for Neuropsychiatric Phenomics dataset is available at https://openneuro.org/datasets/ds000030/. The macaque ECoG data during anesthesia and sleep can be found at http://neurotycho.org/expdatalist/listview?task=78. The QPP template, generated in a previous work26, can be downloaded at https: //github.com/GT-EmoryMINDlab. The human gene expression was from the Allen Human Brain Atlas (https://human.brain-map.org/static/download) and analyzed with abagen toolbox (version 0.0.8, https://abagen.readthedocs.io/en/stable/). Other raw data are not publicly available due to data privacy laws and can be requested from the corresponding author. The data supporting the calculation of hierarchical index are available at Zenodo: https://doi.org/10.5281/zenodo.7855130. Source data are provided with this paper.

Research involving human participants, their data, or biological material

Policy information about studies with human participants or human data. See also policy information about sex, gender (identity/presentation), and sexual orientation and race, ethnicity and racism.

Reporting on sex and gender	We did not consider sex-specific effects in our study, as limited prior research has indicated significant sex differences in the global state of consciousness.
Reporting on race, ethnicity, or other socially relevant groupings	We did not consider socially relevant groupings in our work.
Population characteristics	Dexmedetomidine experiment: 21 healthy male volunteers (age: 26.4 ± 2.1 years; right-handed; body mass index: 21.7 ± 1.9) were recruited from Capital Medical University, Beijing, China.<br><br>Sleep experiment: 6 (out of 22; 1 male/5 females; mean age, 21.6 ± 1.3 years) volunteers with the most consistent sleeping trajectories, as labelled by two experts, were included in the further analyses.
Recruitment	Dexmedetomidine experiment: young healthy volunteers were recruited from Capital Medical University, Beijing, China.To ensure the safety of the experiment, all included volunteers were at an American Society of Anesthesiologists (ASA) physica status I or II.The exclusion criteria included: (1) the presence of metal implants in the body, (2) the presence of intracrania lesions or systemic comorbidities, (3) a history of general anesthesia, (4) a history of drug abuse or alcohol abuse, (5) an allergy to dexmedetomidine,(6)claustrophobia,and/or(7)left-handedness.<br><br>Sleep experiment: young healthy volunteers were enrolled via online advertisement. To ensure their safety, participants with a history of any psychiatric or neurological illness were excluded from the experiment.
Ethics oversight	Dexmedetomidine experiment:: The experiment protocol was approved by the Institutional Review Board of Beijing Tiantan Hospital, Capital Medical University, China. After being informed of the relevant details of the study, all subjects signed written informed consent to their participation.<br><br>Sleep experiment: All experiments were in accordance with the Declaration of Helsinki. The study protocol was approved by the Ethics Committee of Southwest University, China.<br><br>Other datasets used in this study are pre-existing datasets.

Note that full information on the approval of the study protocol must also be provided in the manuscript.

Field-specific reporting

Please select the one below that is the best fit for your research. If you are not sure, read the appropriate sections before making your selection.

☒ Life sciences ☐ Behavioural & social sciences ☐ Ecological, evolutionary & environmental sciences

For a reference copy of the document with all sections, see nature.com/documents/nr-reporting-summary-flat.pdf

Life sciences study design

All studies must disclose on these points even when the disclosure is negative.

Sample size	Our study was validated across multiple independent datasets, and the sample sizes are similar to those in previous works investigating global states of consciousness, such as:<br><br>Three macaques: Barttfeld P, Uhrig L, Sitt J D, et al. Signature of consciousness in the dynamics of resting-state brain activity[J]. Proceedings of the National Academy of Sciences, 2015, 112(3): 887-892.<br><br>The sleep dataset included six healthy participants: Lee M, Sanz L R D, Barra A, et al. Quantifying arousal and awareness in altered states of consciousness using interpretable deep learning[J]. Nature Communications, 2022, 13(1): 1064.<br><br>The anesthesia data consisted of 18 subjects: Sarasso S, Boly M, Napolitani M, et al. Consciousness and complexity during unresponsiveness induced by propofol, xenon, and ketamine[J]. Current Biology, 2015, 25(23): 3099-3105.
Data exclusions	Sleep experiment: Participants with inconsistent sleep stage information labelled by two experts were excluded.
Replication	The hierarchical index was applied to associate with global states of consciousness across different experiments (i.e., sleep, anesthesia, drowsiness, psychedelia, and psychiatric disorders), which verifies the reproducibility of our findings.
Randomization	All participants followed the same experimental protocol, making randomization inapplicable in our study.
Blinding	All participants followed the same experimental protocol, making blinding inapplicable in our study.

Reporting for specific materials, systems and methods

We require information from authors about some types of materials, experimental systems and methods used in many studies. Here, indicate whether each material, system or method listed is relevant to your study. If you are not sure if a list item applies to your research, read the appropriate section before selecting a response.

Materials & experimental systems

n/a	Involved in the study	
☒	☐	Antibodies
☒	☐	Eukaryotic cell lines
☒	☐	Palaeontology and archaeology
☒	☐	Animals and other organisms
☒	☐	Clinical data
☒	☐	Dual use research of concern
☒	☐	Plants

Methods

n/a	Involved in the study	
☒	☐	ChIP-seq
☒	☐	Flow cytometry
☐	☒	MRI-based neuroimaging

Magnetic resonance imaging

Experimental design

Design type	We only consider task-free fMRI across different global states.
Design specifications	Dexmedetomidine-sedation experiment:3 runs (awake, sedation and recovery conditions)
Behavioral performance measures	Dexmedetomidine-sedation experiment: The Observer's Assessment of Alertness/Sedation (OAA/S)scale and Ramsay sedation scale (RSS).

Acquisition

Imaging type(s)	Functional
Field strength	3.0 Tesla
Sequence & imaging parameters	Dexmedetomidine-sedation experiment: fMRI data were recorded using an echo-planar imaging (EPI) sequence in a Siemens Medical Systems Prisma 3.0 T MRI system at Beijing Neurosurgical Institute.The scanning parameters were as follows: TR 2000 ms; TE=30 ms; field-of-view 192 x 192 mm2; acquisition matrix 64 x 64; flip angle 75; slice thickness=4mm; voxel size=3×3×4.4mm3.Sleep experiment: fMRI data were acquired using a 3T Siemens Trio scanner at the Sleep and Neuroimaging Center at Southwest University, Chongqing,China.Head movements were minimized by using a cushioned head fixation device A T2-weighted gradient echo-planar imaging (EPI) sequence was applied, with the scanning parameters as follows: TR 1500 ms; TE 29 ms; field-of-view 192 x 192 mm2; acquisition matrix 64 x 64; flip angle 90; slice thickness =5 mm; voxel size=3×3×5.5mm3
Area of acquisition	Whole-brain

Diffusion MRI ☐ Used ☒ Not used

Preprocessing

Preprocessing software	BRANT (version 3.35)
Normalization	Nonlinear deformation
Normalization template	ICBM 152 Nonlinear Asymmetrical template.
Noise and artifact removal	Regressors included linear trends, averaged signals, and their first-order temporal derivatives within the white matter and cerebrospinal fluid regions, as well as Friston's 24 head motion parameters (3 rotation and 3 translation parameters,6 parameters one time point before.and the 12 corresponding squared items).
Volume censoring	To control the effects of larger motion frames, we conducted an additional scrubbing strategy (FD 0.5 mm) before the nuisance regression and bandpass filtering.

Statistical modeling & inference

Model type and settings	As described in the Method section, mass-univariate test was applied to measure the association between the global state and neural variability.
Effect(s) tested	Spearman's r

Specify type of analysis: ☒ Whole brain ☐ ROI-based ☐ Both

Statistic type for inference<br>(See Eklund et al. 2016)	Whole cortex-wide pattern
Correction	spatial permutation null model was applied to access the significance.

Models & analysis

n/a	Involved in the study
☐	☒ Functional and/or effective connectivity
☒	☐ Graph analysis
☒	☐ Multivariate modeling or predictive analysis

Functional and/or effective connectivity	Functional connectivity was calculated based on Pearson's correlation.
